# Networks of Emotion Concepts

**DOI:** 10.1371/journal.pone.0028883

**Published:** 2012-01-20

**Authors:** Riitta Toivonen, Mikko Kivelä, Jari Saramäki, Mikko Viinikainen, Maija Vanhatalo, Mikko Sams

**Affiliations:** Department of Biomedical Engineering and Computational Science (BECS), Aalto University School of Science, Espoo, Finland; Indiana University, United States of America

## Abstract

The aim of this work was to study the similarity network and hierarchical clustering of Finnish emotion concepts. Native speakers of Finnish evaluated similarity between the 50 most frequently used Finnish words describing emotional experiences. We hypothesized that methods developed within network theory, such as identifying clusters and specific local network structures, can reveal structures that would be difficult to discover using traditional methods such as multidimensional scaling (MDS) and ordinary cluster analysis. The concepts divided into three main clusters, which can be described as *negative*, *positive*, and *surprise*. Negative and positive clusters divided further into meaningful sub-clusters, corresponding to those found in previous studies. Importantly, this method allowed the same concept to be a member in more than one cluster. Our results suggest that studying particular network structures that do not fit into a low-dimensional description can shed additional light on why subjects evaluate certain concepts as similar. To encourage the use of network methods in analyzing similarity data, we provide the analysis software for free use (http://www.becs.tkk.fi/similaritynets/).

## Introduction

A multitude of concepts are used in describing emotional states. The number of such emotion concepts ranges from a few to hundreds, depending on the language and culture [1]. A small child uses a few basic and general emotion categories, but their number steadily increases as the child socializes and his/her capacity to differentiate the world and events becomes more detailed [2]. Similarity of the concepts used to describe emotions varies extensively, ranging from antonyms via concepts having more or less related meanings to synonyms. Various methods have been used to decrease the dimensionality of the “emotion space”. Such reduction is necessary to understand the structure of the mental space underlying the concepts.

A common way to depict the interrelations between emotions or emotion words parsimoniously has been to portray them along two or three orthogonal emotional dimensions. Such description dates back to over a century ago to Wundt [3], who postulated dimensions *pleasant-unpleasant*, *tense-relaxed* and *excitement-depression*. Since then many different dimensional models have been proposed [4], [5], [6]. The most established ones involve as principal dimensions *valence* and *intensity*, where valence refers to the pleasantness *vs* unpleasantness of emotion, and intensity refers to the level of arousal (for a review, see [7]). Early accounts suggested also a *potency-control* dimension, important, e.g., in differentiating emotions sadness and angriness which may be very close in terms of valence and arousal [8]. In their recent extensive study, Fontaine and co-workers [9] added a fourth *novelty-unpredictability* dimension. Recent neuroimaging studies suggest that valence and arousal dimensions may have specific underlying neurophysiological mechanisms [10], [11], [12].

A characteristic feature to emotion concepts is that they form hierarchies of different generality levels. Some concepts are subordinate to others (e.g. cheerful vs. happy). On most general level, emotion concepts divide into a positive and negative cluster, but both these cardinal clusters divide into sub-clusters that then can divide into more specific sub-clusters. Tellegen and co-workers [13], [14] suggested a three-level model, based on factor analyses. The first-order level consisted of discrete emotion concepts, the second-order level of independent positive and negative activation, and the third-order level of bipolar happiness *vs* unhappiness clusters. The model was not hierarchical per se, but it nevertheless depicted emotions on different levels of resolution. Shaver and co-workers [15] and Alvarado [16] used hierarchical cluster analysis to reveal the structure of emotion lexicon. Shaver et al. observed that the clusters at the basic level coincide with a few basic emotions: love, joy, anger, sadness, fear, and possibly surprise. These are the emotions children learn to name first [17]. Alvarado reported two high-level clusters (positive and negative), and eight lower-level clusters (happiness, excitement, lust, melancholy, hate, extreme pain, pain and low-level hostility), into which 135 emotion concepts used in the study divided [16]. The lower-level clusters included subcategories – e.g. joy cluster included passion, ecstasy, arousal, desire and attraction.

Interpreting similarity evaluations between emotion concepts as a weighted network, whose nodes represent the concepts and whose links represent their similarities, we can study them with measures developed specifically for analysing networks. Network theory provides methods for studying the structure of interrelations between elements, from the global level at which the whole network is taken into account, through the intermediate level to the detailed level at which small groups of elements are considered [18], [19], [20]. At a detailed level, motif analysis considers relations between small groups of elements such as triplets. In social networks, interesting motifs include triangles (relations between three individuals) and stars (the relations between a central person and several others) [19], [20]. Our present analyses show that imbalanced triplets are helpful in understanding the similarity evaluations of emotions concepts. At global level, the well-known concept of six degrees of separation [21] iconifies the observation that in social networks the average number of connections that need to be traversed in order to reach a certain element is very small, even in large networks. Another global construction called the spanning tree has been used to identify between different types of epilepsy from EEG data measured from the patient's scalp [22]. Clusters can be considered intermediate-level network features. Real world networks often consist of densely interconnected clusters, which share a function or consist of similar elements [19], [23], [24]. A great effort has been put into devising clustering methods suitable for networks. A handful of algorithms exist for identifying clusters that can share elements [25], [26], [27]. In the present study, we propose using one of these network-based clustering methods and a variant of motif-analysis to increase our understanding of the relations between emotion concepts.

Similarity data on emotion concepts, like many other types of empirical similarity data, often contain patterns that cannot be depicted by any dimensional representation without being distorted. In mathematical terms, triplets in which one distance is larger than the sum of the two shorter distances are said not to satisfy the triangle inequality. They cannot be represented in any metric space, and hence not, for example, in the Euclidean space assumed by dimensional models. One of our aims is to highlight such structures, because their possible presence is a strong motivation for future use of network methods in analyzing this kind of data. Because our data consists of similarities, we would need to convert them to distances in order to determine exactly which concept triplets would not fulfill the triangle inequality. This conversion is not unique and it is thus not uniquely defined where the triangle inequality is not satisfied. Here we simply detect concept triplets with the most imbalanced similarities.

We are still far from understanding the nature of emotions or representation of emotion concepts in mind and brain. Categorical approaches group emotion concepts into subsets so that the concepts within a group share some essential features, and differ from concepts in other groups. The dimensional approaches are useful in revealing a couple of dimensions that explain the structure of the emotion concept space. The present aim was to apply a new method, proved to be successful in understanding phenomena that can be described as networks, to illustrate relationships between emotion concepts at different levels of detail. Importantly, the used method allowed the same concept to be a member in more than one cluster. Our study is also the first to describe mutual relationships of Finnish emotion concepts. Because of the strong influence of culture and language background on emotion concepts [28], such information is a necessary prerequisite for emotion research using native Finnish-speaking subjects.

## Methods

### Stimulus material

Stimuli were 50 commonly used Finnish words describing various emotions, based on a study in which 2020 Finnish speaking subjects were asked to freely produce emotion words [29]. Stimuli were selected out of 57 most commonly produced words, but seven were left out either because they were strongly synonymous with other ones (empathy–*empatia*, desire–*halu*), or because they did not disambiguosly refer to emotional states (hunger–*nälkä*, pain–*kipu*), or were not easily conjugable into the format we wanted (emptiness–*tyhjyys*, fun–*hauskuus*, pride–*ylpeys*). To encourage the subjects to focus on personal experience instead of lexical definition, we presented the concepts as verbs conjugated in first person whenever possible. For concepts that are not naturally expressible as verbs in Finnish, we used adjectives with the instruction to include “I feel …/ I am …” (“*Olen* …”) with each adjective.

The list of the used words is shown in Table 1, ordered by the frequency of their occurrence [29]. The English translations grasp the meanings of the words relatively well, although an exact translation using only one or a few words is impossible. For some words, we give two translations. In the figures, we use only the part typed in boldface.

**Table 1:** 50 Finnish emotion concepts used in the experiment, and their English translations.

1	vihainen	angry	26	myötätuntoinen	compassionate
2	iloinen	cheerful	27	epävarma	insecure/uncertain
3	rakastan	feeling love	28	epäilen	doubting/suspicious
4	surullinen	sad	29	apea	melancholic
5	pelkään	afraid	30	hellä	tender
6	onnellinen	feeling happiness	31	huolestunut	worried
7	kateellinen	envious	32	säälin	feeling pity
8	ahdistunut	distressed/anxious	33	ärtynyt	irritated
9	väsynyt	tired	34	helpottunut	relieved
10	masentunut	depressed	35	luottavainen	trusting
11	tuskainen	tormented	36	mustasukkainen	jealous of attention
12	ihastunut	having a crush on	37	suuttunut	angered
13	tyytyväinen	content	38	turhautunut	frustrated
14	inhoan	disgusted	39	häpeän	ashamed
15	jännitän	nervous	40	levoton	restless
16	pettynyt	disappointed	41	raivostunut	enraged
17	kaipaan	missing/longing for	42	rohkea	courageous
18	rauhallinen	calm	43	hämmästynyt	surprised
19	ikävöin	pining for/missing	44	himoan	feeling lust for
20	toiveikas	hopeful	45	kauhuissani	terrified
21	katkera	resentful	46	innostunut	excited
22	riemastunut	overjoyed	47	ihmettelen	wondering
23	välinpitämätön	indifferent	48	hämmentynyt	confused
24	epätoivoinen	despairing	49	onneton	unhappy/miserable
25	tylsistynyt	bored	50	anteeksiantava	forgiving

### Subjects and data collection

20 native voluntary Finnish-speaking subjects (university students or colleagues; age range 19–31 years; 10 female, 10 male) were asked to quantify on the scale 0–5 how similar they perceive the emotional states represented by pairs of emotion concepts. Verbal descriptions of the values were: 0) not similar at all; 1) very weakly similar; 2) weakly similar; 3) moderately similar; 4) very similar; 5) extremely similar. We instructed the subjects to base their evaluation on personal experience rather than on dictionary definitions of the terms. Each word pair was shown on a computer screen, and the subject could indicate the similarity by pressing a corresponding number on the keyboard. At any time only one pair was visible. However, it was possible to return to the previous pair and re-evaluate it if the subject wanted to correct the evaluation. Subjects rehearsed with five word pairs, including very similar and very dissimilar pairs, with words not included in the experiment. These test pairs were aimed to help the subjects to calibrate their similarity scale. The experiment was carried out according to the principles of Declaration of Helsinki. The data were analysed anonymously. When the experiment was done, Helsinki University of Technology, now Aalto University, did not have an Institutional Review Board to give ethical approvals for behavioral experiments. However, the experiment was totally non-invasive and was evaluated by the experimenters to be ethical and harmless to the participants. A verbal consent was regarded to be appropriate for this kind of experiment. It was emphasized to the subjects that they can finish doing the experiment if they feel like that.

Fifty words can be combined into 50×49/2 = 1225 pairs. 120 of the pairs were presented twice to assess stability of the evaluations by the same subject. The same pairs were shown twice for each subject. We selected from a preliminary data of two subjects 40 pairs with similarity above 3, 40 pairs with similarity between 0 and 3, and 40 pairs with zero similarity. Each subject thus gave 1345 similarity evaluations in a session that took approximately two hours. The experiment was done in four parts, between which the subject could take a break if he/she wished. The experiment was done in a soundproof room with minimal distractions. Stimulus pairs were presented in a random order that was different for each subject. Moreover, within each pair the word order was random, such that some subjects would see the pair *cheerful–hopeful* and others the pair *hopeful-cheerful*.

After the similarity evaluations, we asked the subjects to rate each of the 50 emotion concepts on three features: valence, intensity, and interactivity. The extremes of each scale were described verbally. For valence (V), we used the scale −3–3 (integers), from “a very negative emotion for me” to “a very positive emotion for me”. For intensity (I), we used a scale 0–6, from “a very calm emotion” to “a very strong emotion and a high state of alertness”. For interactivity (IA), we also used the scale 0–6, from “other people are not relevant for this emotion” to “other people are highly relevant for this emotion”. Subjects also filled the 20-item Toronto Alexithymia Scale (TAS-20) questionnaire for identifying alexithymia [30]. Alexithymia is a personality trait characterized, among other things, by difficulty in identifying and describing feelings.

### Conversion of similarities to distances

For the multidimensional scaling analysis, we converted our similarity data to distances using a linear transformation. Any distance measure should be zero between an element and itself, and nonzero between any other pair. Our similarity scale from 0 to 5 did not include a value to represent identity, the similarity of an element to itself. For a meaningful conversion to distances, we needed to discern identical pairs from highly similar pairs, and hence we needed to extend the scale. We chose the next integer value (6) to represent identity. We then converted the similarities *s* to distances *d* linearly, *d = 1−s/6*, such that concept pairs with zero similarity are said to be at distance 1, and the distance between a concept and itself becomes zero. All other similarity values were transformed to distances between zero and one. With this kind of conversion in multidimensional scaling short distances are well preserved, in contrast to e.g. *d = 1/s* (*d = 50*, when *s = 0*), which stresses long distances. The averages over subjects were calculated for *s*, so distances *d* were always average distances over all subjects.

### Multidimensional scaling (MDS)

Multidimensional scaling (MDS) methods are designed for data that consists of dissimilarities or distances *d* between pairs of *n* data elements. They reduce the data to a small number *k* of dimensions, attempting to keep similar elements close to each other, and dissimilar elements far from each other. Two-dimensional scaling, like the one used in this study, enables a visual representation of the pattern of distances amongst a set of elements in a plane. MDS finds a set of vectors, representing the elements in 2D-space, such that the matrix of their Euclidian distances is as similar as possible with the original distance matrix *D* according to a certain criterion. There are many possible criteria, but we chose to apply metricstress:
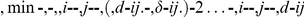


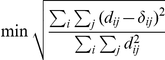
Here ,*d-ij.* are the pairwise distances in the original 50-dimensional space and ,*δ*-Here 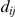 are the pairwise distances in the original 50-dimensional space and 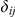 are the distances of the vectors in the new 2D-space. MDS is robust in terms of the choice of the stress criterion [31].

MDS methods can discover underlying factors that explain the similarities, analogously to what factor analysis finds from feature data. The meaning of the found dimensions is an interpretation of the experimenter and not objective. Unless the data actually has the same number of dimensions as the MDS representation, the similarities or distances will be distorted. However, if the distance data is based on a small number of (at least ordinal-valued) features, multidimensional analysis can reveal these features or dimensions.

### Networks and their visualization

A *network* consists of elements, called *nodes*, and of connections between the nodes, called *links*. The strength of a connection can be depicted by the *weight* of the link. The similarity data produced by each of our twenty subjects can be interpreted as a weighted network, in which the nodes are the fifty emotion concepts, and the weighted links correspond to the nonzero similarities between concepts. This network has *N* = 50 nodes and the number of links (nonzero similarities) must lie between 0 and *N*(*N*−1)/2 = 1225.

A network visualization can display similarities between all concept pairs. Furthermore, node properties can be displayed by color or shape. A multitude of information can thus be combined in a single figure. In the network plots in this article the similarity of each concept pair with *s*>0 is shown as a link. The length of a link does not have a meaning; the degree of similarity is displayed by link color and width. Because the layout is produced by a dimension reduction method, similar concepts tend to be placed near each other, and hence thick red links tend to be short.

*Thresholding* a network means discarding links up to (or below) a given weight. Thresholding was used for finding out groups of highly similar elements (clusters of concepts with similar meaning).

For visualizing networks, we need to specify coordinates for the nodes. A suitable layout will guide to observe the essential structures of the network. If the layout of nodes is generated with an appropriate dimension reduction method, the visualization can also reveal possible underlying dimensions similarly to a multidimensional scaling plot. Because network visualizations can display link weights and node properties, all information is displayed, but the choice of coordinates affects the readability of the information. Various dimension reduction algorithms could be used to determine coordinates, and there is no unique or ‘correct’ way to do it. We will use methods that are known by experience to work well. For visualizations of networks, we determined node coordinates using the graph-drawing software Himmeli [32]. The algorithm constrains the nodes into two dimensions using a dimension reduction method in which springs of different stiffness are placed between node pairs according to their weight (similarity). The minimum energy of the spring system is sought, such that similar nodes will be placed close to each other and dissimilar nodes further away (length of a link is meaningless). Node properties can be displayed by color or shape. A multitude of information can thus be combined in a single figure.

### Cluster analysis

A large variety of *cluster analysis* methods exist for grouping elements into clusters whose members are ‘similar’ or ‘close’ to each other in some respect [23], [33]. In complex networks theory, cluster analysis is usually called community detection, in reference to clusters in social networks. *Hierarchical clustering methods* attempt to detect group hierarchy, such that a cluster may contain several smaller clusters, which may further contain smaller clusters. So the structure is nested with different levels of detail.

In weighted networks, one way to determine cluster hierarchy is to generate networks consisting of successively stronger links by discarding links up to various weight thresholds, and detect clusters in each thresholded network. We thus obtain clusters consisting of successively stronger links. For detecting clusters at each threshold level, we employed a method called *clique percolation* (CP; [27]). An advantage of using the CP method is that it allows a node to belong to more than one cluster, in contrast to other clustering methods that have to our knowledge been used for studying the interrelations of emotion concepts. For example, in our data the concept *feeling love* may belong both to a positive *love* cluster (together with *having a crush on* and *feeling happiness*) and to a negative *love* cluster (together with *missing* and *pining for*). In addition, the method does not force every node to belong to some cluster, but allows nodes to remain isolated.

**Figure 1:**
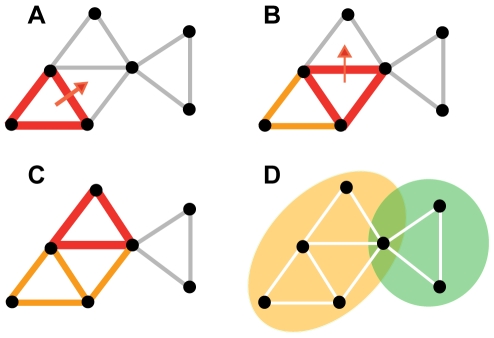
The SCP method. For detecting clusters of similar concepts, we use the SCP method, defining clusters as sets of adjacent triangles. The triangle shown in red can be rolled between five nodes in two steps (a→b→c), such that at most one node changes place at each step. These nodes constitute the cluster marked in orange in d). The three nodes marked in green form a separate cluster. The definition allows a node to belong to more than one cluster. In this example, the green and orange clusters overlap through a shared node.

Technically, CP defines a cluster as a group of nodes within which every node can be reached by ‘rolling a clique’, as depicted in Fig. 1. Any clique (fully connected subgraph) could be used, but because our 50-node network is rather small, we will use only triangles. The method is deterministic, meaning that every time it is run on a specific network, the same clusters will be detected. The processes of thresholding the network at every possible level and detecting clusters according to the CP method in each thresholded network has been combined into an algorithm called *sequential clique percolation* (SCP; [34]). The SCP algorithm produces a tree plot called a *dendrogram* that shows how clusters break into smaller clusters when the threshold level is increased.

### Statistical significance of triangles

Because our clusters are defined on the basis of adjacent triangles, it is important to make sure that the triangles are statistically significant. We test against the null hypothesis that the similarities observed in our data are randomly placed between concept pairs. This kind of structure would result from shuffling the pairwise similarity evaluations. At each threshold level, the pairwise similarity values drawn from the null model (corresponding to our null hypothesis) form a network, whose structure (ignoring weights) is fully random, a so-called Erdös-Rényi network [35]. This is because all similarities in the null model are placed between random concepts, and because thresholding removes a random subset of them. At each threshold, a certain number of link weights (similarities) exceed the threshold. This equals the number of edges in the random network.

We can assess the statistical significance of triangles observed in the original network by comparing their number against the expected number of triangles in random null model networks by using the Z-score,
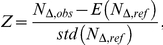
where 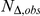 is the number of triangles in the original network, 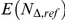 is the expected number of triangles in the random null model and 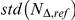 the corresponding standard deviation. For random networks 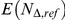 and 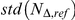 can be calculated analytically (see [36]).

Instead of the E-R model, network scientists commonly use the configuration model, where structural correlations are removed from networks by rewiring their links while keeping their degree sequences intact. Although applying the configuration model would first appear to be a natural choice for the analysis in this paper, the E-R model can be considered a better alternative here, since there is no ensemble of distance matrices for which the thresholding process would produce the sequence of network ensembles produced by the configuration model approach. To verify our results, we have despite this also applied the configuration model to the data; the results are qualitatively similar to the ones presented in the Results section for the E-R-model.

### Imbalanced triplets

Intuitively, if concepts A and B are similar, and concepts B and C are similar, then A and C ought to be fairly similar as well. Our intuition is based on everyday experience of dealing with dimensional spaces. Similarity data is not necessarily dimensional, however. Our data contains many concepts triplets that break the pattern, such as *love*–*missing*–*forgiving*, in which the pairs *love*–*missing* and *love*–*forgiving* are evaluated on average as highly similar, but the similarity of the third pair *missing*–*forgiving* is judged to be very low. Such concept triplets show that similarity evaluations are not based on fixed features of concepts. Instead, the context set by each pair may affect their similarity evaluation. The similarity relations within these triplets are difficult to assess with dimensional analysis, because the distances (dissimilarities) between the concepts do not fulfill the triangle inequality and hence may be heavily distorted in a dimensional space.

### Software

We provide an online software package for analyzing any similarity data using the main methods introduced in this section: thresholding and visualization of networks, finding hierarchical clustering structure with sequential clique percolation and locating imbalanced triplets. The software is free and publicly accessible from the URL: http://www.becs.hut.fi/similaritynets/.

## Results

### Description of the data

**Figure 2:**
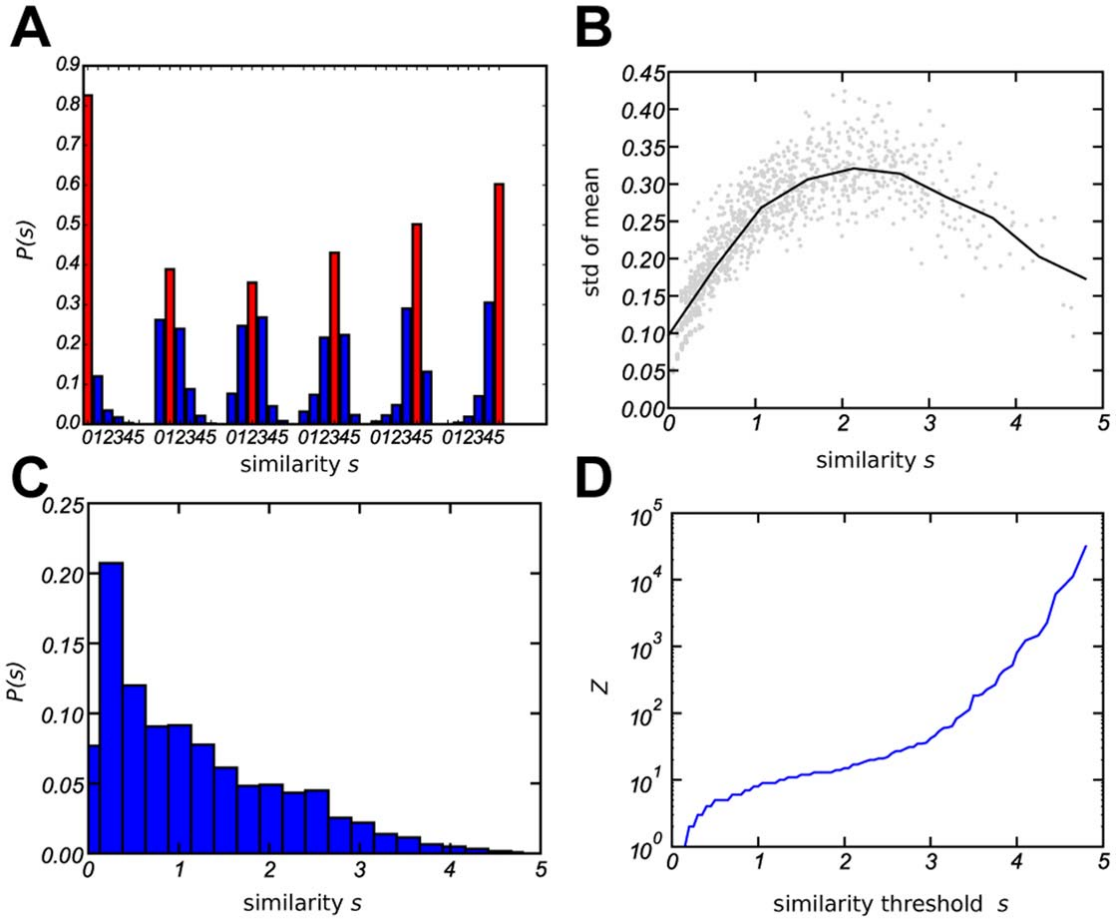
Statistics of the similarity data. (A) Variations observed when the same concept pair was evaluated twice. The six histograms have been constructed using each repeated pair. Red indicates the frequency *P(s)* of identical evaluations from most dissimilar (0) to most similar (5). Blue bars indicates the frequency of non-identical evaluations, which were typically just above or below the other evaluation. (B) Variability of averaged similarities. Each dot represents a concept pair, such that the horizontal axis indicates the mean similarity *s* of the pair averaged over all evaluations and the vertical axis its standard deviation. The solid curve follows an average of all points with a given similarity. (C) The similarity distribution *P(s)* of the Average Similarity Network (ASN). (D) The *Z*-score of the observed number of triangles as a function of similarity threshold, calculated using the null hypothesis of random networks with the same number of links.

Subjects were not fully consistent when evaluating the similarity of the same concept pair twice, roughly 40% of the evaluations differed. Histograms in Fig. 2A show the fraction of the evaluations that were the same (red bars) or different (blue bars), when the similarity evaluation varied from 0 (left-most histogram) to 5 (right-most histogram). Each repeated pair contributes twice, because we did not distinguish between the ordering of evaluations. Subjects were most consistent in evaluating highly similar or dissimilar concept pairs, being somewhat more inconsistent with in-between similarity values. Importantly, the differences were quite small, the second evaluation being mostly one unit lower or higher than the first one.

We can only speculate about the possible reasons for inconsistencies. A subject might feel the appropriate similarity is not well described by an integer, but with a decimal number like 2.5, which could be rounded either to 2 or 3 with equal probability. Importantly, similarity evaluations of concept pairs do not occur in isolation, but preceding evaluations may evoke recollections that influence forthcoming evaluations.

When similarity data are averaged over all 20 subjects (Average Similarity Network, ASN), the variability of evaluations becomes moderate (Fig. 2B). Each dot in Fig. 2b represents a concept pair. The horizontal axis indicates the mean similarity of a pair, and the vertical axis the standard deviation of the mean. The solid curve displays an average of all points at a given mean similarity *s*. Concept pairs with low or high values of similarity have the smallest variance (the standard error of the mean is roughly 0.1 for similarities close to zero). Even the largest standard deviation of mean, about 0.3, is still not very large compared to our similarity scale, which ranges from 0 to 5.

The distribution of evaluated similarities is shown in Fig. 2C. Only a small fraction of the concept pairs were evaluated as very similar (about 2% have *s*≥3.5). Most of the concept pairs were evaluated to be quite dissimilar.

The number of triangles observed in the thresholded ASN is hugely larger than expected in a network of the same link density with random structure. The Z-score in Fig. 2D reflects the statistical significance of the observed triangles in the ASN (see Methods). It increases rapidly as a function of the similarity threshold *s*. Already for *s* = 1, the observed number of triangles is roughly 10 standard deviations larger than in the null hypothesis, and for s = 4 the difference is on the order of 1000 standard deviations. This shows that the triangles observed, and hence the clusters we detect in the ASN, are extremely unlikely to arise by chance.

### Average Similarity Network (ASN)

**Figure 3:**
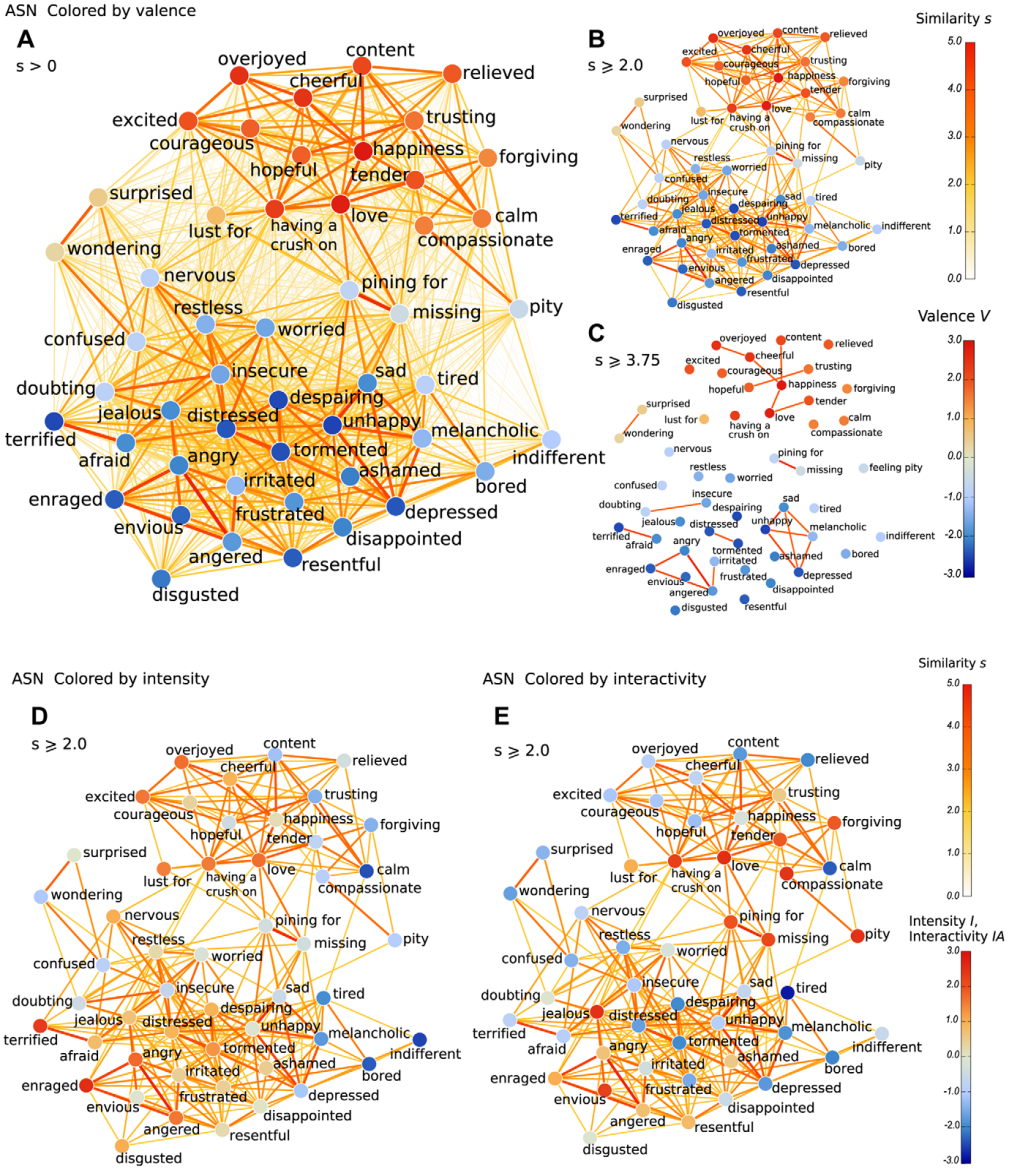
Visualizations of the Average Similarity Networks.

In ASN, all 50 concepts are interlinked with a vast number of connections (Fig. 3A). Every concept is linked to nearly every other concept, with at least very low similarity links. Because the low-similarity links are faded to the background using pale colors, we nevertheless see that the structure divides into regions of negative valence (blue) and positive valence (red). Displaying only similarities *s*≥2.0 (thresholding the network) reveals the division into two main clusters more clearly (Fig. 3B). The positive and negative cluster are bridged mainly by connections between the love-related positive concepts *love*, *tender*, and *having a crush on* on the one hand, and negative concepts related to longing and restlessness, namely *pining for*, *missing*, *restless*, and *nervous* on the other. A somewhat separate group of concepts related to surprise (*surprised*, *wondering*, *confusion*) stands out. Certain concepts have very few connections to others (e.g. *indifferent*, *lust for*). Two sub-clusters containing both positive and negative valences are also discernible, namely *love – pining for – missing*, and *forgiving – compassionate – pity*. When the ASN is thresholded at a higher similarity value, such that only links with s≥3.75 remain (Fig. 3C), two strong sub-clusters of the main negative valence cluster related to anger and unhappiness are revealed, as well as several highly similar concept pairs.

Intensity corresponds slightly less clearly to network structure than valence (Fig. 3D). Both the positive and negative clusters contain regions with high and low intensity (Fig. 3D), and the low-intensity concepts are mainly located on the right. An exception are the concepts related to confusion (*surprised*, *wondering*, *confused*, *doubting*, *insecure*). Interactivity does not change smoothly in any direction of the two-dimensional layout, but local structures are rather homogeneous in interactivity values (Fig. 3E). The main positive cluster has a highly interactive part of love-related concepts (*having a crush on*, *love*, *tender*, *forgiving*, *compassionate*), and a very mildly interactive part. The former connects to the highly interactive negative valence concepts *pining for*, *missing*, *and pity*. Most of the concepts in the main negative cluster are not very interactive. The highly interactive negative concepts *envious* and *jealous*, describing social emotions, are located among moderately interactive concepts.

### Individual similarity networks

Some subjects perceived fewer or lower similarities between concept pairs than others. Although all 20 subjects used the whole similarity scale from 0 to 5, they employed different regions of the scale in different amounts, and the average similarities differed greatly. The mean similarity of individual subjects, averaged over all possible pairs of the 50 emotion concepts, ranged from 0.22 to 2.57, reflecting strong individual differences. The average mean similarity over all 20 subjects was 1.15±0.65 (mean ± std). There was large variation in the link density of networks of individuals within each gender group. We did not find a statistically significant difference in the number of nonzero links for the two gender groups, (two-way Welch's t-test of group means, independent pairs, possibly unequal variances, estimated degrees of freedom 17.3, t(17.3) = −2.0, p = 0.058).

**Figure 4:**
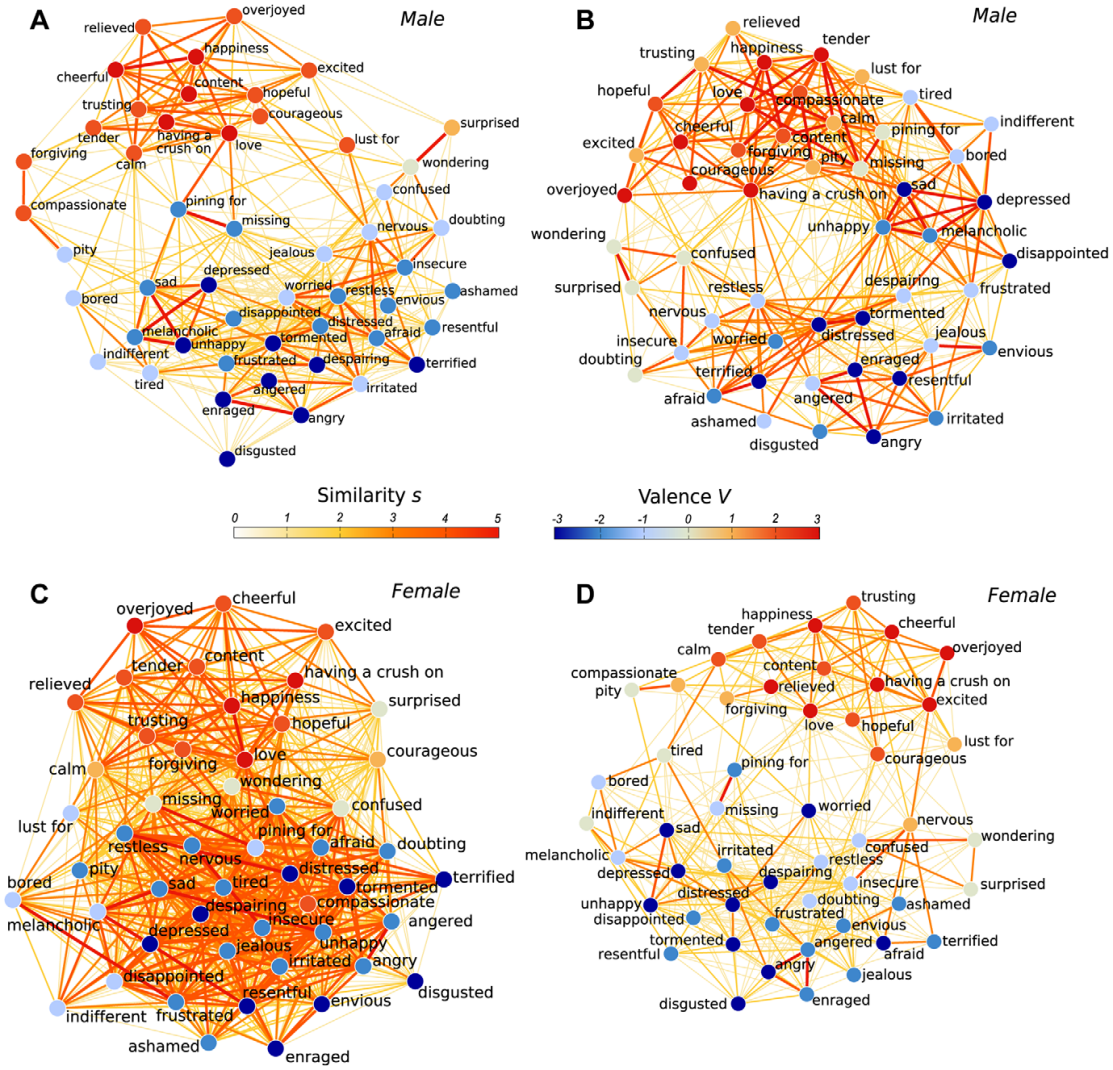
Similarity networks of two male and two female subjects (s>0).

We illustrate the differences between individual subjects' data with four examples (Figs. 4A–D). The different link densities discussed above are visible. Moreover, slightly different clusters and connections stand out in the four individual networks. For example, in the network of a male subject (Fig. 4A), the positive and negative clusters are very distinct, whereas in the network of another male subject (Fig. 4B) the positive cluster is strongly linked to sadness-related negative concepts. The network in Fig. 4B contains a relatively distinct cluster of anger and jealousy, whereas in Fig. 4A jealousy is not very strongly connected to anger but rather to insecurity and doubt. Moreover, in the network in Fig. 4B the concept *having a crush on* has unusually many and strong connections to negative emotions, including *restless* and *angered*, compared to the other individuals' networks (based on visual inspection of the data). In the network of a female subject (Fig. 4C) connections are so dense and strong that sub-clusters cannot be distinguished without clustering algorithms. The positive and negative clusters are discernible, but they have many strong interconnections. In contrast, the network of another female subject (Fig. 4D) has unusually few strong similarities.

### Hierarchical cluster structure

**Figure 5:**
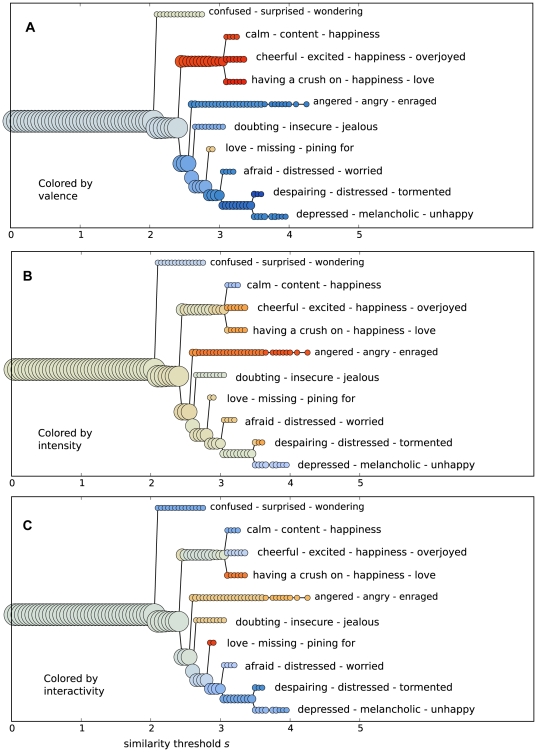
Cluster hierarchy of the Average Similarity Network.

*Dendrograms* that depict the cluster hierarchy of the average similarity network ASN (N = 20) are depicted in Figs. 5A–C. The area of each disc indicates cluster size (number of words in the cluster). The similarity thresholds, which indicate the minimum similarity values that were retained and used to construct the clusters, are displayed on the horizontal axis. Each horizontal branch corresponds to a cluster, and the forking of a branch indicates that the cluster breaks into smaller clusters.

The network divides into three main clusters. The first two coincide well to the subjects' categorization of the concepts to **positive** and **negative**. The third cluster is a distinct ***surprise*** (*confused, wondering*) cluster (Fig. 5A). The positive cluster divides into three sub-clusters ***calm*** *(content, happiness)*, ***cheerful*** *(excited, happiness, overjoyed)*, and ***love*** (*having a crush on, happiness*). Interestingly, *happiness* is in each of these clusters. The negative cluster divides into six sub-clusters ***angered*** (*angry, enraged*), ***doubting*** (*insecure, jealous*), ***missing*** (*loving, pining for*) ***afraid*** (*distressed, worried*), ***despairing*** (*distressed, tormented*), and ***depressed*** (*melancholic, unhappy*).

The sub-clusters of the positive and negative main clusters differ in their average intensity (Fig. 5B). The most intense sub-clusters are those related to anger, cheerful, and love. The least intense ones are those related to surprise, calm, and depressed. The average interactivity values of each sub-cluster also vary greatly. The most interactive clusters are those related to love, both positive and mixed (Fig. 5C). The doubt and anger cluster are relatively interactive as well. Interestingly, intensity and interactivity seem to be features that can differentiate meaningfully the positive-valence cluster to three separate sub-clusters.

The end of a branch in Fig. 5 indicates the highest threshold at which the cluster still exists. Above this threshold, some crucial links of the cluster are removed and the cluster dissolves. For example, the cluster *confused*, *surprised*, *wondering* breaks apart from the rest of the network roughly at *s* = 2.1 and dissolves at *s* = 2.8, i.e. the branch extends from threshold 2.1 to 2.8. A long branch means that a cluster is strong in relation to nearby concepts. In other words, many of the concepts within the cluster are more similar between themselves than to other concepts. The further right the branch ends, the stronger the links within the cluster are. At the end of each branch are the concepts in the cluster just before the cluster dissolves. A slice of the dendrogram at threshold *s* corresponds to the clusters detected in the network thresholded at *s*.

### Multidimensional scaling, the valence-intensity plane, and the SCP cluster division

**Figure 6:**
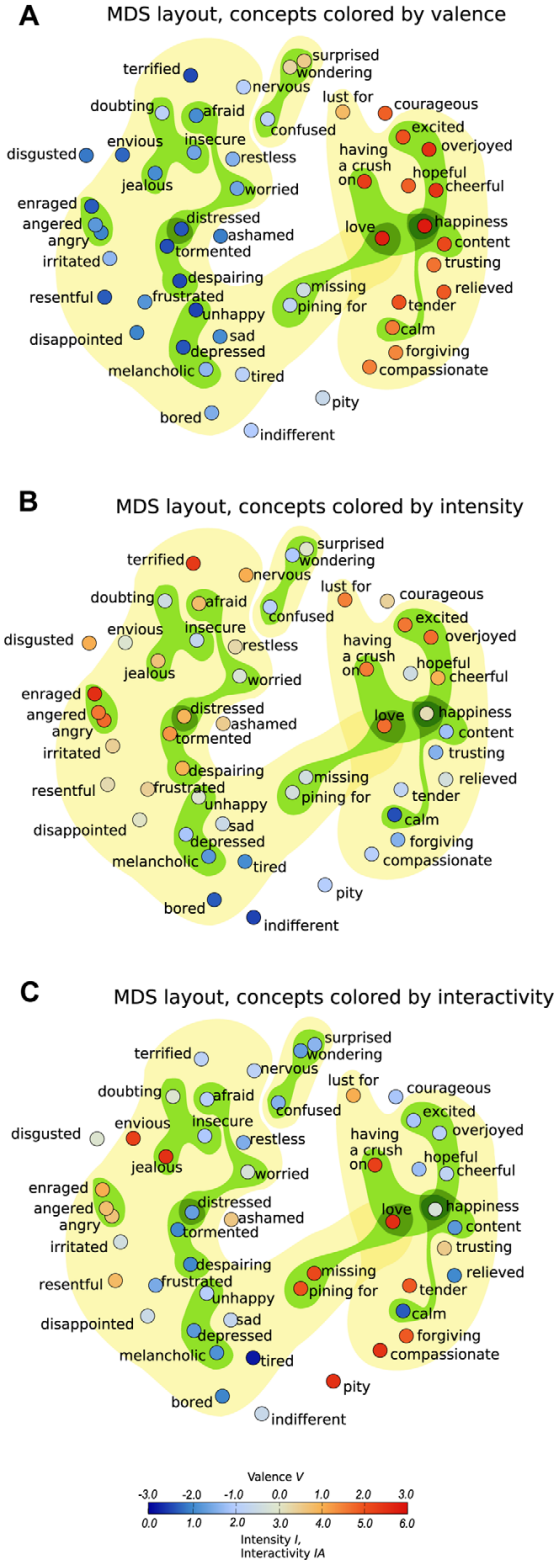
Layout of emotion concepts based on a two-dimensional scaling of the similarity data averaged over all subjects. Each concept is colored by the subjects' averaged evaluation of its (A) valence, (B) intensity, or (C) interactivity. Highlighted regions correspond to clusters determined by the SCP cluster analysis method. The smaller green regions each depict a leaf of the dendrogram. The three larger yellow regions correspond to clusters at the highest hierarchy level at which the largest cluster has been divided into a positive, negative and surprise cluster (similarity threshold *s* = 2.45).

To compare the cluster structure and a dimensional description of the same similarity data, we analyzed the data using multidimensional scaling (MDS). Figure 6 displays the two-dimensional MDS layout, with concepts colored by valence, intensity, and interactivity. The coloring lets us verify visually how the discovered dimensions relate to these characteristics. The rotation of the coordinates is arbitrary, and the resulting layout varies slightly each time the MDS analysis is done on the same data. The valence evaluations (Fig. 6A) shift fairly smoothly from blue (negative valence) on the left, to red (positive valence) on the right, verifying visually that a dimension of valence is in good agreement with the structure of the data. An exception is the concepts that were perceived to be the most negative – *unhappy (V = −2.5)*; *despairing (V = −2.45)*; *terrified (V = −2.45)*; and *tormented (V = −2.4)* – which are not located furthest to the left. This demonstrates the difficulty of reducing a network of relations into a few dimensions. The intensity evaluations (Fig. 6B) shift slightly less smoothly than valence from blue (low intensity) at the bottom right, towards red (high intensity) at the top left. Relatively low intensity concepts related to confusion and uncertainty (*confused*, *wondering*, and *insecure*) are located among high intensity concepts in the top left region, and a few high intensity concepts, *excited*, *overjoyed*, and *feeling love*, are placed next to low intensity concepts. Despite these deviations, intensity appears to be a relevant dimension in explaining the structure of similarities. Interactivity explains the two-dimensional layout clearly worse than valence and intensity.

Now, let us compare the clustering division against the dimensional representation (node locations). In Fig. 6, the cluster division obtained by SCP is highlighted in color, so that the yellow regions correspond to clusters at the highest hierarchy level (similarity threshold *s* = 2.45) and the green regions correspond to leaves of the SCP dendrogram, which represent the longest-lasting clusters. Node coloring by valence, intensity and interactivity shows at a glance how homogeneous the clusters are in these characteristics. If we attempted to observe groups of concepts based on their location, assuming the most similar concepts to be grouped close by in the dimensional layout, several categories that stand out in the SCP cluster division would escape us. Although MDS is not meant for detecting categories, it is informative to observe what kind of relations between concepts it ignores that can be revealed by clustering analyses. For example, the MDS layout does not hint that *surprise* could be strongly separate from the negative and positive emotion concepts. We would also not observe the mixed-valence subcluster *pining for–missing–love*, an intuitively meaningful cluster related to longing. Some concepts (*disgusted*, *courageous*, *indifferent*, *pity*, and *ashamed*) are so dissimilar to the other ones that they do not participate in any cluster. Furthermore, the positive subclusters related to *love*, *intense joy* and *calm* would have been difficult to discern, as well as certain negative subclusters such as *afraid–worried–distressed*. In sum, a division into clusters finds concept groups that could not be deduced from the two-dimensional MDS plot alone. Using a large number of dimensions might be helpful here, but many-dimensional layouts are difficult to visualize and interpret.

### Imbalanced triplets and the valence-intensity (V-I) plane

Certain concepts, such as *love* and *happiness*, participate in several clusters in the hierarchy produced by SCP. Closer inspection of the similarity data shows that these concepts are often evaluated as very similar to two other concepts, which themselves are evaluated as fairly dissimilar. The existence of such imbalanced triplets may hinder dimensional reduction analysis, where the dissimilarity between two constituents of such a triplet is difficult to balance against the similarity of these concepts to the third constituent.

**Figure 7:**
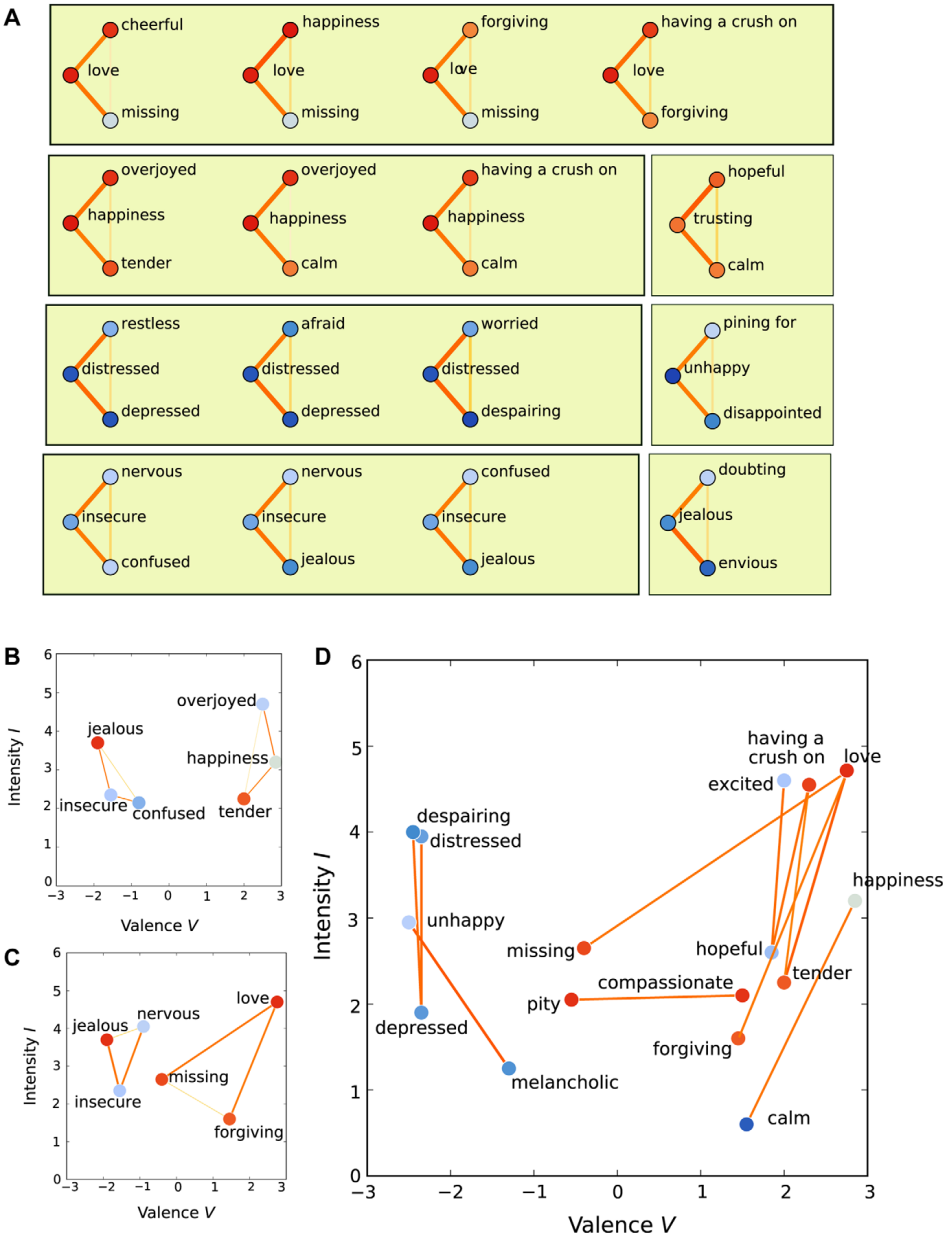
The imbalanced triplets. (A) The most imbalanced 16 concept triplets, in which one concept is perceived very similar to two others, whereas these two are perceived very different. The concepts are colored by their mean valence evaluations. The similarity between each concept pair is shown by link color and width, using color scales as in Fig. 3. (B) Two examples of imbalanced triplets shown on the valence-intensity (V-I) plane, where the coordinates are from mean valence and intensity evaluations of the emotion concepts. In these examples, the central concept is located between the dissimilar concepts. The concepts in panels B–D are colored by interactivity, using color scales as in Fig. 3. (C) Two more examples of imbalanced triplets on the V-I plane, where the least similar pair turns out to be located closest in the V-I-plane. (D) Concept pairs with strong similarities (*s*≥3.0) that are distant in the V-I-plane (Euclidian distance *d*≥2.0).

Figure 7 displays the most imbalanced triplets in ASN, and highlights the triplets and concept pairs that are most at odds with the valence-intensity plane. For Figure 7a, we selected triplets where 1) the two stronger similarity (*s_1_*, *s_2_*) values were ≥3.0 and the weakest similarity was at most half of either of the two stronger similarities (*s_3_*≤min(*s_1_*, *s_2_*)/2). These limits singled out the most imbalanced 16 triplets. The central concept in these triplets was most often *love*, *happiness*, *distressed*, or *insecure*. *Love* and *happiness* are broad concepts, and the triplets expose their multiple connotations or contexts in which they appear. For example, we see that happiness is related to intense joy (*overjoyed*), relaxation (*calm*) and love (*having a crush on*), which are also visible in the hierarchical clustering structure (Fig. 5). Similarly, the imbalanced triplets centered on *distressed* and *insecure* highlight different connotations of these concepts.

For some triplets, the central concept was roughly located between the other two in the valence-intensity plane; two such examples are displayed in Figure 7B. This is in line with the representation of emotion concepts in valence-intensity space. For some of the other imbalanced triplets, however, the most dissimilar connection was not explained by the location of the concepts in the valence-intensity plane (Fig. 7C). For example, in the triplet *love–missing–forgiving* the weakly similar concept pair is the least distant in the valence-intensity plane.

Previously it has been noted that there exist concept pairs with similar valence and intensity values having different meaning. Complementary to this, Figure 7D shows concept pairs that were perceived to be similar (*s*≥3.0), but were nevertheless distant in the valence-intensity plane (Euclidian distance *d*≥2.0) determined by the evaluations of the concepts by the subjects. The vertical trend of these deviations shows that these pairs often had similar valence, but for some pairs neither valence nor intensity explains the similarity. In particular, *missing* differs from *love* both in valence and in intensity, but the concepts are perceived as very similar. Probably being a mixed emotion that is connected to the emotions of sadness and love, *missing* does not fit easily into the dimensional depiction. In contrast, it fits well in a categorization where clusters are allowed to share concepts, as is seen in the hierarchical cluster structure (Fig. 5). It turns out that nearly all of these pairs are congruent in interactivity (depicted by node color in Figs. 7A–D).

## Discussion

In this paper we suggest new methods for analyzing relations between emotion concepts. Responding to the observation that emotional categories are not mutually exclusive [37], we suggest using a hierarchical clustering method that allows concepts to belong to several clusters. Moreover, we suggest comparing similarities between concept triplets for identifying concepts that adopt diverse meanings depending on context, and are hence most likely belong to several clusters. These methods of motif-analysis and network clustering come from network theory [18], [19], [20]. They are well suited for studying similarities between emotion concepts, which constitute a weighted network. Adding to studies of emotion categories across cultures [15], [38], [39], [40], [41], this study provides a first look at the cluster structure of Finnish emotion concepts.

Using a hierarchical clustering method based on similar concept triplets [27], [34], we identified the strongest concept groups, or cores within the fuzzy categories. Earlier studies on the hierarchy of emotion concepts have found five basic categories: *love, happiness, anger, anxiety/fear*, and *sadness*, constant across the languages of American English, Indonesian, and Basque [15], [38], [41]. We observe somewhat similar structures: *Happiness*, *anger*, and *sadness* form clearly distinct categories in our cluster tree. A moderately strong *distress/fear* subcluster is discernible from a larger *distress/sadness* cluster. Our set of 50 concepts contained fewer words than the studies [15], [41], which naturally affects the categories that can arise.

In contrast to earlier results, we see a relatively strong cluster related to *jealousy/doubt*. Love is placed as a subcategory of happiness; the large happiness-related cluster subdivides into clusters of *love*, *cheerfulness*, and *contentedness*. There are many possible explanations for the different cluster structures. They could be simply different views of the same hidden, fuzzy structure of emotion categories. They could be in part due to cultural dependencies as well. Subcategories are quite culture-dependent [40], [41]. In our data, the relatively strong category related to *jealousy/doubt* could be a cultural specificity, but testing this would require more data. The small number of concepts in our study, as well as the limited number of subjects, unfortunately limits the comparisons we can make. Finally, the clustering methods differ. In contrast to the clustering method used in [15], [41], the SCP method we use [34] will not place a concept in a cluster if it is only weakly similar to others. This way, SCP identifies only the clearest subgroups. Moreover, SCP allows concepts to belong to more than one cluster. This allows us to see a mixed valence subcluster related to *longing*, containing the concept *love* that also appears in a positive context in the happiness cluster.

In many studies of hierarchical clustering of emotion concepts, subjects have been asked to divide the given concepts into meaningful groups [15], [16], [41]. Our experimental setup has the advantage that using similarity data, we do not guide subjects to consider grouping or hierarchy. Nevertheless, we obtain a strong hierarchical cluster structure. When the concepts are not forced into distinct categories, relations that do not fit a crisp categorization can be revealed.

The standpoint that a small set of emotions is more basic than others has strongly been argued for [42] and against [43]. When subjects are asked to group emotion concepts into categories, the level of detail in the grouping will range from very broad to very detailed [15], and asking people to list subcategories of an emotion concept such as anger reveals that “people do not share an explicit, ready-made, well-elaborated taxonomy for types of anger” [37]. There is no consensus as to how many subcategories there should be, or what they should contain. Emotion concepts can be meaningfully categorized at different levels of detail. The coarsest-level (superordinate) categories of emotion concepts correspond to positive valence, negative valence and surprise. At the subordinate level, cultural differences have been observed [41]. As is evident also in our data, there are very strong individual differences in the use of emotion concepts.

A cluster related to surprise stands out early on from the positive and negative clusters in our cluster hierarchy, similarly as in the clustering of English emotion concepts by [15]. This early distinction marks surprise as a very different emotion from the positive and negative ones. Ortony and Turner [43] take the position that surprise is not an emotion because an affective state should be valenced in order to be called an emotion, but many others have considered surprise as one of the fundamental or basic emotions. If emotions are seen as mechanisms developed during the evolution of our species that activate the proper responses to a given situation [44], [45], surprise can be seen as a fast occurring emotion that primes us to evaluate the situation and decide whether it is positive or negative. Characteristic features of surprise are a high arousal level and short duration.

We compared the hierarchical categorical representation and a dimensional depiction of the structure of emotions, which has a long tradition [3], [4], [5], [6], [7], [9]. The two-dimensional layout produced by multidimensional scaling corresponded very closely to subjects' evaluations of valence, and moderately to their evaluations of intensity. It turns out that most subclusters are relatively homogeneous in valence, intensity and interactivity. Intensity can discern among some of the positively valenced clusters (and among some of the negatively valenced ones), but not all. In some cases, situational and relational content (such as interactivity) rises above the valence and intensity as factors in determining high similarity. The concepts that do not seem to fit the dimensional view, such as mixed-valence emotions (*longing*), can be better accommodated with a categorical model with overlapping clusters.

Several researchers have suggested that similarity judgments of emotions are based on comparing properties of emotions [46]. Studying similarities between concept triplets gives us hints of the basis of similarity evaluations. Identifying such triplets is a version of motif-analysis tailored for this particular data; for a review of motif-analysis see [47]. If similarity evaluations were based on absolute, unchanging attributes of the emotion concepts, and similarity was judged on the same attributes for each pair (see [48], for a discussion of this view), we should not see imbalanced triplets such as *calm*, *happiness*, *overjoyed*, for which two pairs (*happiness* -*calm* and *happiness-overjoyed*) are seen as highly similar, but one (*calm-overjoyed*) is not considered similar at all. The presence of imbalanced triplets shows that two words set a context, which determines their similarity (cf. [37]).

### Limitations of our study and ideas for future research

Only twenty subjects were used for this study, aimed at testing whether the network methods could reveal relations between emotion concepts that are difficult to detect using earlier methods. A larger set of subjects would make the findings more reliable, and also allow us to compare the networks of men and women, and describe individual differences. A larger set of emotion words would give us a better view of the relations between Finnish emotion concepts, and allow better comparison with cluster structures in other languages. A drawback of using similarity evaluations instead of asking subjects to group the concepts is that we cannot use very large sets of words. An experiment with 50 word pairs already takes about two hours. The similarity evaluations for a larger set of words could also be collected by dividing the set of concept pairs across several subjects, but then individual differences would be lost.

Individual differences in the structure of emotion knowledge could possibly be used in understanding how e.g. persons with Asperger syndrome understand and use emotion concepts. Such persons have specific difficulties in understanding social emotions [49], [50]. Our subjects completed the 20-item Toronto Alexithymia Scale (TAS-20) questionnaire [30], so that we could attempt to correlate their TAS score with network characteristics. In earlier studies, a network characteristic (the diameter of a spanning tree) has been found to identify the type of epilepsy from EEG measurements [22]. We could not reliably study spanning trees or path lengths of individual networks, because the similarity evaluation of a particular concept pair could have a large error, leading to errors in these measures. The measures we considered, such as the number of nonzero links, or the valence, intensity, and interactivity evaluations of each concept, did not correlate significantly with TAS. Our relatively small number of subjects and small range of TAS scores may prevent discovering a possible correlation with some structural measure of the similarity network and the TAS-scores.

Although our clustering method allows overlapping clusters, it cannot account for the full networked structure of concepts. Russell and Fehr found in their study [37] that categories are not necessarily ordered: A can be a subcategory of B and B a subcategory of A simultaneously. Our method (or any hierarchical clustering method for that matter) does not allow for such recursive categories. A complete picture of the fuzzy structure of emotion categories is very difficult to depict. The SCP method extracts an approximation, and as with any clustering method, there are limitations as to what it can depict. It is however one of the few hierarchical methods allowing for overlapping clusters [25], [26], [34].

The category approach has its rightful place along the dimensional approach in representing emotional concepts. As the boundaries of categories are inherently fuzzy and overlapping, they can better be represented with a hierarchical clustering method that allows overlapping clusters. The proposed methods help to assess broad emotion concepts or mixed emotions, and to place them in a hierarchical representation.
